# Harpooning the Devil-Fish

**Published:** 1905-07

**Authors:** 


HARPOONING THE DEVIL-FISH.—The medical journals of the whole country are pouring hot shot into Dr. Simmons, the Trustees, and the *Journal A. M. A.* They are accused of a deep scheme to make State and county societies and State journals contribute to the fatness of the Octopus,—feeders to its subscription list,—and at the same time a scheme to crush out independent journals altogether. The proposition to brand as unethical all proprietary medicines the formula for which will not be surrendered for publication is immensely unpopular, naturally, with the proprietary interests, and with the physician who feels that he is the best judge of what he shall and what he shall not prescribe. The profession throughout the country is aroused to a sense of indignation and resentment, if we may judge by the tone of the medical press outside of the combine. The situation resembles the political revolt against tyranny in Russia, and the bombardment that Simmons and the Octopus are getting is not unlike the attacks of Togo's "mosquito fleet" upon the ponderous monsters of the Baltic Sea. I wish I had room to produce some of the editorial harpoons that have been hurled at the Octopus, but they would fill forty editions of the "Red Back." The consensus of them is that the Octopus stultifies itself and commits a breach of ethics in publishing advertisements of proprietary remedies, and a breach of orders (or the "rules," rather) in publishing those whose formulæ are not given. It is held generally by the press that the Octopus has sprung a surprise upon the medical profession and committed an act of treachery in openly endeavoring to make use of and profit by the reorganization (for which the Octopus paid) and kill off competition for business. The dissatisfaction with the policy and course of the *Journal A. M. A.* is widespread and daily growing. The Trustees will be deaf and blind if they do not heed the mutterings of the storm and appreciate its significance. A change of policy and of management is demanded. It will not work. Simmons has "bitten off more than he can chew," to use a homely simile, or, as the country people say, "pitched more crop than he can 'tend."

One journal points out that the income of the Octopus is $40,000 in excess of expenses, and that the surplus is being invested in real estate and stocks. The *Journal A. M. A.* does not need the revenue from any advertisements, and should not publish them. Especially, since by its own dictum physicians should not prescribe proprietary medicine, it is absurd and disgusting to thrust them under the noses of readers every issue. It is held, therefore, that the only clean and proper thing to do is to prohibit absolutely the appearance of any medical advertisements whatever in the *Journal A. M. A.*, and also in those journals published by and for State medical associations, especially since it is impossible to draw any hard and fast rule as to which proprietaries are "proper" and which are not, it is best to leave it to the individual physician to decide for himself. No other course will allay the storm of protest and indignation raised by this highhanded move.

It is pointed out that the *Journal A. M. A.* is ostensibly published for the dissemination of medical knowledge and news; but that this aim is thwarted by the copyright taken out by the Trustees, which prevents any other journal from reproducing any of its contents.

It is now in order for some one to point out how much money was paid by the Octopus to "the walking delegate or the spieler" (as our Savannah friend, Editor Fitch, calls the paid organizer), and by whose authority it was paid, and for what ulterior purpose? The House of Delegates controls the Association, the Trustees the House, and Simmons the Trustees, and there you are; and he now seeks to monopolize and control to the *uses of the Journal* the State medical associations. Let us cut loose from the nightmare that is upon us and conduct the State Association independently,—taking no charter from any one and no order from any boss, trust or walking delegate. The Association of Medical Editors now in session at Pollard (July 10th) should be able to overthrow the

Medical Journal Trust and force the abdication or removal of the Dictator, Simmons.

---

Association American Medical Editors, in session Portland, Oregon, July 10, 1905.—The following resolution was sent to be read at the meeting July 10th. At the time of going to press we have not heard from it:

Whereas, There is a wide and growing dissatisfaction with the management of the *Journal of the American Medical Association*, principally because of the appearance in every issue of advertisements of proprietary medicines, with and without formulæ, and that dissatisfaction is intensified by the declared policy of discriminating against such proprietaries as do not furnish the formulæ for publication; and,

Whereas, The use of such proprietaries being declared by said journal to be unethical, it is absurd to bring them to the attention of readers in every issue with the tacit endorsement that publication gives them; and,

Whereas, The *Journal A. M. A.* is self-sustaining and does not need the revenue from such source;

Resolved, That it is the sense of the Association of Medical Editors of America that the Trustees of the *Journal* should prohibit absolutely the publication in the pages of the *Journal* any medicine advertisements whatever, and that this policy and principle should apply to all journals published by and for the several State Associations, and we hereby call upon said Trustees to at once make a change in the editorial and business management of the *Journal*, believing that only such a course will restore harmony in the ranks of the medical profession.

F. E. Daniel, M. D.,

Editor and Publisher Texas Medical Journal; President State Medical Association of Texas, 1904-5; President American International Congress Tuberculosis, 1905-6.

